# Prevalence and characterization of pain in radiation oncology: the PREDORT multicenter cross-sectional study

**DOI:** 10.1007/s12094-024-03603-4

**Published:** 2024-07-30

**Authors:** Fernando Arias, Uxúe Zarandona, Berta Ibáñez-Beróiz, Reyes Ibáñez, Maider Campo, Jon Cacicedo, Noelia García-Rueda, Beatriz Baztán, Raquel Villanueva, Marta Fresán, Iñaki Redín, Ana T. Osés, Victoria Hurtado, Inés Villafranca, Vasti Iancu, Pilar Almeida, Nieves Moreno, Soraya Cadena, Irene Carruesco, Marián Allegue, Ana B. González, PREDORT Collaborative Group

**Affiliations:** 1 Radiation Oncology Department, University Hospital of Navarre, Irunlarrea 3, Pamplona, Spain; 2 University Hospital Miguel Servet, Saragossa, Spain; 3 University Hospital of Álava, Vitoria, Spain; 4 University Hospital of Cruces, Bilbao, Spain

**Keywords:** Pain and radiotherapy services, Oncologic pain, Pain prevalence

## Abstract

**Background:**

Pain in cancer patients has enormous impact on their quality-of-life. Radiation therapy (RT) is a cornerstone in cancer treatment. The objective of the PREDORT study is to estimate the prevalence of pain in patients attending at Radiation Oncology (RO) Services.

**Methods:**

A prospective, multicenter study was designed for patients treated at the RO Services of reference hospitals. Patients were seen in their initial Nursing consultation, during which key data was collected, including demographic and comorbidities data, medical history, and oncological and pain characteristics. The study has received approval from the Ethics Committee of Navarra, and all patients signed the Informed Consent.

**Results:**

Of the 860 participating patients, 306 reported some type of pain, which implies a prevalence of 35.6%. Of them, 213 identified a cause of oncological origin. The proportion of pain was similar among sexes, but the proportion of non-cancer pain was higher among women (*p* < 0.05). Regarding pain intensity, the magnitude of breakthrough pain in patients with oncological pain is nearly 1 point greater than in patients with non-oncological pain (7.53 vs 6.81; *p* = 0.064). Cancer pain is more likely to be limiting of normal life than non-cancer pain (59% versus 38%, *p* < 0.001). Regarding analgesic treatment, only 60/306 patients (19.6%) were receiving strong opioids. There were 68 patients with pain without any treatment (22.2%).

**Conclusions:**

The prevalence of pain in cancer patients referred to RO services is 35.6%, with the prevalence of exclusively oncological pain being 24.8%. Understanding and addressing oncological pain is essential to provide comprehensive care to patients.

## Introduction

Pain in cancer patients is a common and highly significant symptom due to its enormous impact on their quality of life [1]. The reported prevalence of pain in these patients ranges from 20% to 60% [2]. Variations in pain prevalence rates in oncology can be explained by factors such as the proportion of patients with localized or metastatic disease, cultural differences in pain perception, and methods used to determine these prevalence rates [3].

Radiation therapy (RT) is a cornerstone in cancer treatment, either exclusively or in combination with surgery or chemotherapy (including hormone therapy and the latest targeted therapies), and can be administered with curative or palliative intent. It is estimated that approximately 60% of cancer patients will require radiation therapy at some point [4, 5]. Understanding and addressing oncological pain is essential to provide comprehensive care to patients and enhance their overall well-being.

Oncologic pain can have multiple causes and manifest in different forms, such as acute or chronic pain, and can be caused by the tumor itself, metastasis, medical procedures, or therapy side effects [6], not to mention other non-oncologic causes.

Very few published data exist regarding the real and current prevalence of pain in Radiation Oncology services, with only one article [7] featuring a sufficient number of patients (> 1000), albeit exclusively analyzing neuropathic pain. The objective of the PREDORT study is to estimate the prevalence of pain in patients attending at Radiation Oncology Services and to characterize its types, in order to provide useful information for therapeutic management.

## Materials and methods

A cross-sectional multicenter study was designed for all patients treated at the Radiation Oncology services of reference hospitals (University Hospital of Navarre—UHN, University Hospital of Álava—UHA, Miguel Servet University Hospital from Zaragoza—MSUH, and Cruces University Hospital from Bilbao—CUH) over a period of approximately 2–6 months, depending on the center, which had agreed to participate. Assuming that 40% of the sample suffered from pain, a sample size of *n* = 850 was required to obtain a precision of ± 3% for the 95% confidence interval of the estimate for prevalence of pain. This would give a precision of ± 5% for hospitals with *n* = 400 participants and a precision of ± 10% for hospitals with about *n* = 100 participants.

Patients were seen in their initial Nursing consultation—previously they were particularly trained—during which key data were collected, including demographic and comorbidities data, medical history, and oncological and pain characteristics. Pain variables included presence of pain, current level intensity (analog visual scale), tumor-related pain, duration of pain, location, presence of breakthrough pain, triggers, whether pain limits daily activity and variables related to pain treatment. Demographic, medical history, and oncological data were obtained from medical records, co-morbidities from patient-reported data verified by reviewing the medical history, and pain data from primary patient-reported data.

In respect of pain intensity in oncologic patients, we have followed the suggestions of the majority of cancer studies, which recommend a cut-off point equal to 4 in the Visual Analogical Scale (VAS) for mild–moderate pain and either equal to 6 or 7 for moderate-severe pain [8].

The study has received approval from the Ethics Committee of Navarra, and all included patients have signed the appropriate Informed Consent.

### Statistical analysis

Sociodemographic, clinical, and pain-related patient characteristics were described using means and standard deviations, or frequencies and percentages, for both the total sample and each participating center.

According to the revised IASP definition of pain [9], we have defined pain as an unpleasant sensory and emotional experience associated with, or resembling that associated with, actual or potential tissue damage. Pain prevalence was estimated through proportions, along with a 95% confidence interval using the Wilson method. Comparison of pain characterization variables between different pain types was done using the chi-squared test or Fisher's exact test for categorical variables as applicable, and *t*-tests or Mann–Whitney *U* tests for continuous variables, depending on the distribution type. The statistical analyses were performed using IBM SPSS 21 and openepi software programs.

## Results

This study included a total of 860 patients, with a median age of 64.5 years (21–97 years). 50.1% were male. General characteristics of the patient cohort are presented in Table 1. A proportion of 79.2% of patients received Radiation Therapy with curative intent, while 20.8% received it with palliative intent. The most frequently treated tumor types were breast cancer (33%), followed by uro-gynecological tumors (24.6%), lung cancer (16.1%), head and neck cancer (9.3%), digestive cancers (8.5%), and others. Most patients (66.5%) were non-smokers, whereas a total of 18.2% were smokers and 16.3% were former smokers. A total of 10.1% of patients reported others previous primary tumors, and over 87% of patients had associated comorbidities, with more than 67.1% having > 2 comorbidities. At the Miguel Servet University Hospital, comprising 17.4% of the study population, there was a lower proportion of lung cancer cases and a higher proportion of uro-gynecological cancers, leading to a lower rate of smokers in that group. RT was curative in 681 patients (79.2%), and palliative in 179 (20.8%).

**Table 1:** Descriptive characteristics of the total sample and by hospital

	Total	UHN	UHV	MSUH	CUH
Patients; *n* (%)	860	497 (57.7%)	190 (22%)	150 (17.4%)	23 (2.6%)
Age; mean (SD)	64.5	64.3	64.2	65.9	59.8
Sex					
Men	432 (50.1%)	252 (50.2%)	93 (48.9%)	80 (53.3%)	7 (30.4%)
Female	428 (49.6%)	245 (49.8%)	97 (51.1%)	70 (46.7%)	16 (69.6%)
Tumor type					
Breast	288(33.0%)	160 (32.2%)	72 (37.9%)	41 (27.3%)	13 (56.5%)
Lung	138(16.1%)	97 (19.5%)	27 (14.2%)	13 (8.7%)	1 (4.3%)
Gyn-Uro	211 (24.6%)	116 (23.3%)	49 (25.9%)	46 (30.7%)	0 (0%)
Digestive	73 (8.5%)	44 (8.9%)	17 (8.9%)	12 (8%)	0 (0%)
H&N	80 (9.3%)	38 (7.8%)	12 (6.3%)	21 (14.0%)	9 (39.1%)
Others	70 (8.0%)	42 (8.5%)	13 (6.8%)	17 (11.3%)	
Smoking status					
Active	147 (18.2%)	88 (18.9%)	34 (17.9%)	17 (11.3%)	8 (34.8%)
Ex smoking	123 (15.3%)	106 (22.8%)	14 (7.4%)	2 (1.3%)	1 (4.3%)
Non smoking	536 (66.5%)	271 58.3%)	129 (67.9%)	122 (81.3%)	14 (60.9%)
Comorbidities					
Yes	750 (87.2%)	431 (86.7%)	174 (91.6%)	132 (88%)	13 (56.5%)
No	110 (12.8%)	66 (13.3%)	16 (8.4%)	18 (12.0%)	10 (44.5%)
RT type					
Radical	681(79.2%)	379 (76.3%)	155 (81.6%)	124 (83.7%)	21 (91.3%)
Palliative	179 (20.8%)	118 (23.7%)	35 (18.4%)	24 (16.3%)	2 (8.7%)

*UHN* University Hospital of Navarre; *UHV* University Hospital of Vitoria; *MSUH* Miguel Servet University Hospital; *CUHC* Cruces University Hospital

Of the 860 participating patients, 306 reported some type of pain, leading to a prevalence of 35.6% (95% CI 32.4, 38.8). Of them, 213 identified a cause of oncological origin (caused by the tumor or by oncological treatments), which implies that 24.8% of the total participants had cancer pain (95% CI 22.0, 27.8).

Pain characteristics of the patients that reported pain are reflected in Table 2. Patients whose pain had an oncological origin—identified by the nurses according to the answers of patients—reported a shorter duration of pain than patients whose pain has another origin (*p* < 0.001). Likewise, of the patients who had cancer pain, 77.9% reported that their pain was continuous, while for those who had not cancer pain, only 55.9% stated that it was continuous (*p* < 0.001). The proportion of patients that reported breakthrough pain was 47.2%, without significant differences by oncological or not oncological type (*p* = 0.330).

**Table 2:** Profile of type of pain and comparison among characteristics by kind of pain

	Total (*n* = 306)	Kind of pain		*p*-Value
		Non-oncological (*n* = 93)	Oncological (*n* = 213)	
Pain duration				
< 3 months	148 (47.5%)	24 (26.4%)	119 (56.7%)	< 0.001
> 3 months	158 (52.5%)	67 (73.6%)	91 (43.3%)	
Continuous pain				
No	88 (28.8%)	41 (44.1%)	47 (22.1%)	< 0.001
Yes	218 (71.2%)	52 (55.9%)	166 (77.9%)	
Breakthrough pain				
No	161 (52.8%)	53 (57.0%)	108 (50.9%)	0.330
Yes	144 (47.2%)	40 (43.0%)	104 (49.1%)	
Magnitude				
VAS continuous pain	5.59 (2.09)	5.44 (2.01)	5.65 (2.13)	0.491^a^
VAS breakthrough pain	7.90 (1.89)	6.81(2.03)	7.53 (1.93)	0.064^a^
Activity limitation				
No	145 (47.5%)	58 (62.4%)	87 (41.0%)	< 0.001
Yes	160 (52.5%)	35 (37.6%)	125 (59.0%)	

Missing data: 5 patients had missing data for pain duration; 1 for breakthrough pain; 1 for activity limitation

^a^Mann–Whitney *U* test

Regarding pain intensity, breakthrough pain was higher in magnitude according to the VAS scale than the continuous pain, and further, the magnitude of breakthrough pain in patients with oncological pain was nearly one point greater than in patients with non-oncological pain (7.53 vs 6.81; *p* = 0.064). Finally, according to the self-reported-data by the patients, cancer pain is more likely to be limiting of normal life than non-cancer pain (59% vs 38%, *p* < 0.001).

A comparison of the proportion of pain by sex is made in Table 3. The proportion of patients reported pain was similar between both sexes (37% in women vs 34% in men), but the proportion of non-cancer pain was higher among women than among men, with a prevalence equal to 12.8% (95%CI 10.0, 16.3%) compared to 8.8% (95%CI 6.5, 11.8) in men (*p*-value = 0.028).

**Table 3:** Proportion of pain and type of pain by sex

		Total (*N* = 860)	Pain	Oncologic pain	Non-oncologic
Female	*N* *p* (95%CI)	428 (49.8%)	159 37% (32.7, 41.8)	104 24.3% (20.5, 28.6)	55 12.8% (10.0, 16.3)
Male	*N* *p* (95%CI)	432 (50.2%)	147 34% (29.7, 38.6)	109 25.2% (21.4, 29.5)	38 8.8% (6.5, 11.8)
Female vs male *p*-value			0.340	0.376	0.028

Regarding analgesic treatment, of the 306 patients with pain, only 60 patients (19.6%, 95%CI 15.5–24.4) were receiving opioids of the third step of the WHO [10], while 238 patients (77.7%, 95%CI 72.8–82.1) were receiving only first or second-step analgesia (see details in Table 4). Of note, there were 68 patients with pain but without any medical treatment (22.2%, 95%CI:17.8–27.2).

**Table 4:** Analgesic use at the moment of the interview

Patients with pain (*N* = 306)	Treatment	Proportion (95%CI)
First step analgesics	238	77.7% (72.8, 82.1)
Second step analgesics	36	11.8% (8.6, 15.9)
Third step analgesics	60	19.6% (15.5, 24.4)
No drugs	68	22.2% (17.8, 27.2)

*Patients could be on multiple treatments

## Discussion

Our study showed a pain prevalence estimate of 35.6% (95%CI 32.4, 38.8) among patients treated at the Radiation Oncology Services of four Spanish reference hospitals, and an oncological pain prevalence of 24.8% (95% CI 22.0, 27.8), without not relevant variations between most hospitals. General patient characteristics were similar among the highest-recruiting centers. However, at the Cruces University Hospital (CUH), accounting for 2.7% of the study patients, the majority had breast cancer, which usually does not involve oncological pain. This population was notably younger with fewer comorbidities. This was likely due to the fact that only specific conditions were treated at that medical consultation, explaining the lower rate of palliative radiotherapy in that center also showed that, considering those patients that reported pain, the proportion of participants with continuous pain was high (71.2%), especially among those that reported oncological pain (77.9%), who also reported activity limitations with higher frequency (59%), In our study, the prevalence of pain was similar in all hospitals but HUC (which corresponds to only the 2,7% of the sample), and ranges between 36.6% and 40% (Table 5, Appendix) are similar to those from other series with similar characteristics [11].

**Table 5:** Pain characteristics according to the different hospitals in the study

	Total	HUN	HUA	HUMS	HUC
Having pain?					
No	554 (64.2%)	315 (63.4%)	114 (60%)	110 (73.8%)	14 (60.9%)
Yes	306 (35.6%)	182 (36.6%)	76 (40%)	39 (26.2%)	9 (39.1%)
Duration					
< 3 months	148 (48.3%)	85 (80.5%)	35 (46.1%)	–	8 (88.9%)
> 3 months	158 (51.7%)	97 (19.5%)	41 (53.9%)	–	1 (11.1%)
Pain type					
Oncological					
Tumor	176 (57.5%)	105 (21.1%)	43 (56.6%)	26 (66.7%)	2 (22.25)
Treatment	76 (24.8%)	33 (6.6%)	22 (29.3%)	15 (38.5%)	6 (66.7%)
Unknown	23 (7.5%)	21	1	1	0
Non oncológical	130 (42.5%)*	125 (69.8%)	33 (43.4%)	23 (59.3%)	7 (77%)
Pain intensity (EVA)					
Continuous	5.57	5.6	5.8	5.1	4.3
Irruptive	7.3	7.77	6.6	7	N/A
Limits activity	160 (52.5%)	108 (59.7%)	34 (44.7%)	17 (43.6%)	1 (11.1%)

*Several patients have both pain types, oncological and non-oncological

The study is the first published article on pain prevalence in Radiation Oncology, aside from smaller studies like Jamora et al. [12], with less than 100 patients, and Mañas et al. [7], which focused solely on neuropathic pain. In Jamora single institution study, they noted a high prevalence (62.8%) of pain in 94 oncologic patients undergoing radiotherapy. Among these, the majority experienced mild pain (67.8%) with low pain interference (67.8%) on day-to-day personal life. Mañas et al. study was carried out more than fifteen years ago, and they found a high prevalence (31%) of neuropathic pain among cancer pain patients visiting RT oncologic units.

Certain cancer types are highly sensitive to radiation, and it can be used alone to shrink or eliminate tumors or radical RT [13]. In other cases, radiation may be administered before surgery to reduce tumor size (neoadjuvant therapy) or after surgery to decrease the chance of recurrence (adjuvant therapy). Radiation therapy can also be used palliatively, especially for advanced cancers, to alleviate problems like pain, swallowing difficulties, breathing issues, or intestinal blockages [14]. Acute (e.g., acute mucositis) and chronic (e.g., radiation-specific chronic pain syndromes) pain can also arise as side effects of radiation [15]. This suggests that the real prevalence of pain may be slightly higher due to radiation-related pain, but it could be balanced by patients experiencing relief, particularly those with bone metastases with radiotherapy treatment [16, 17].

Regarding pain duration, the fact that more than 50% experienced pain for over three months is noteworthy, even taking into account that this proportion goes down to 43% in the case of oncological pain. Additionally, over 50% of patients with breakthrough pain had a known trigger, implying room for improvement in its treatment [1]. Despite numerous guidelines and protocols [18, 19], only about 20% of patients with any kind of pain received third-step analgesic treatment, significantly lower than recommended [20]. This may be due to a certain refusal on the part of Spanish doctors to prescribe these drugs considered first choice for intense pain, especially of oncological origin. In this sense, it could be interesting to know if it is the patients with most pain who receive higher dose of opioids, but this date is out of the scope of this study.

Following WHO recommendations [21], about pain intensity in oncologic patients (cut-off point equal to 4 in the Visual Analogical Scale (VAS) for mild-moderate pain and either equal to 6 or 7 for moderate–severe pain, our results for continuous pain, with a VAS score equal to 5.6, suggest that our patients have moderate intensity of pain, whereas those for breakthrough pain, with a VAS score equal to 7.3, have severe pain, which seems to agree with other patients’ cohorts [22].

It is important to account that pain prevalence and pain intensity alone do not illustrate completely the problem underlying cancer pain, since interference of pain with daily activities need to be considered. In fact, more than 50% of patient with any kind of pain in our series referred interference of pain with daily activities. Although this analysis is outside the scope of this study, the topic has been assessed in depth by other authors, such as Te Boveldt et al. [23, 24]. They showed that having reported worst pain in the last week was associated with having reported pain interference with daily activities, and that the greater the intensity of the pain, the greater the impact on daily activity. Nevertheless, other studies have showed that there are also patients that report low intensity of pain, but still it has great impact on their daily life [25], which suggest that pain and its influence on daily activity may vary depending on other features which go beyond intensity.

Gender's influence on pain perception is of interest, with notable higher rates of non-oncological pain among females, aligning with existing literature [26].

Another point of interest is the rate of comorbidities. Effectively, the rate of comorbidity found in this cohort of patients was high (87.2%), but similar to the rest of population, and the rate of smokers and ex-smokers was higher, probably due to the presence of a high proportion of lung cancer patients.

Considering that over 50% of cancer patients will receive radiation therapy either alone or in combination with other treatments, it can be inferred that prevalence rates of pain found in this study could be applicable to the broader oncology population.

The primary study limitation is the potential bias in patient selection due to limited participation of healthcare staff in some centers despite the substantial sample size. Also, the study does not allow a distinction between neuropathic and other types of pain. Furthermore, since it is an analysis only at the beginning of radiotherapy treatment, the study does not allow you to analyze the evolution of pain throughout the course of treatment.

However, the study strengths lie in its large sample size, particularly at the Hospital Universitario de Navarra, where most patients were included.

## Conclusion

According to the study, the prevalence of total pain in cancer patients referred to radiation oncology services (35.6%), although lower than previously reported, it is still high with the prevalence of oncological pain being 24.8%. Of the patients with pain in our study, 22.3% received no analgesic treatment, even though 52.5% stated that their activities of daily living are impaired and more than 50% experienced pain for more than 3 months. This data justify solid training of radiation oncologists and other specialists in analgesic treatment taking into account that “of the patients with pain in our study, 22.3% received no analgesic treatment, even though 52.5% stated that their activities of daily living are impaired and more than 50% experienced pain for more than 3 months” and these patients were seen by other specialists before entering into radiation oncology service.

## Data Availability

Not applicable.

## Appendix

See Table 5.
